# Computer vision enables short- and long-term analysis of *Lophelia pertusa* polyp behaviour and colour from an underwater observatory

**DOI:** 10.1038/s41598-019-41275-1

**Published:** 2019-04-29

**Authors:** Jonas Osterloff, Ingunn Nilssen, Johanna Järnegren, Tom Van Engeland, Pål Buhl-Mortensen, Tim W. Nattkemper

**Affiliations:** 1 Biodata Mining Group, Faculty of Technology, Bielefeld University, PO Box 33501, Bielefeld, Germany; 2 Equinor, Research and Technology, 7005 Trondheim, Norway; 3 Norwegian Institute for Nature Research, P.O. Box 5685 Torgarden, 7485 Trondheim, Norway; 4 NIOZ Royal Netherlands Institute for Sea Research, Department of Estuarine and Delta Systems, Utrecht University, Korringaweg 7, 4401NT Yerseke, the Netherlands; 5 Research Group Benthic Habitat, Institute of Marine Research, 5817 Bergen, Norway; 6 Department of Biology, Ecosystem Management Group, University of Antwerp, Universiteitsplein 1, 2610 Wilrijk, Belgium

**Keywords:** Image processing, Marine biology

## Abstract

An array of sensors, including an HD camera mounted on a Fixed Underwater Observatory (FUO) were used to monitor a cold-water coral (*Lophelia pertusa*) reef in the Lofoten-Vesterålen area from April to November 2015. Image processing and deep learning enabled extraction of time series describing changes in coral colour and polyp activity (feeding). The image data was analysed together with data from the other sensors from the same period, to provide new insights into the short- and long-term dynamics in polyp features. The results indicate that diurnal variations and tidal current influenced polyp activity, by controlling the food supply. On a longer time-scale, the coral’s tissue colour changed from white in the spring to slightly red during the summer months, which can be explained by a seasonal change in food supply. Our work shows, that using an effective integrative computational approach, the image time series is a new and rich source of information to understand and monitor the dynamics in underwater environments due to the high temporal resolution and coverage enabled with FUOs.

## Introduction

Because of their high biodiversity and importance as habitat for fish, the cold-water coral (CWC) reefs receive much attention from academia and authorities as well as NGO’s and the general public^1,2^. CWC habitats are commonly found in deep waters, often far from shore, and accessing these systems is challenging. Despite the challenges, CWC in the North East Atlantic and US-waters are reasonably well studied, on shorter time scales. In the NE Atlantic, the stony coral *Lophelia pertusa* is the dominant CWC reef builder^3,4^, forming large three-dimensional structures that provides habitat for many other species. In addition to its function as biological hotspot, CWC reefs also play an important part in biogeochemical cycles and processing of organic matter in the deep-sea^5–7^.

Forty years ago, knowledge on geographical distribution of CWC reefs was only based on reported by-catch from fishermen, in addition to a limited number of surveys^3^. Active knowledge gathering provided by academia and industry has traditionally been based on physical sampling by dredging and/or grabbing^8–10^. Such sampling methods are destructive for the sampled organisms and the habitat. Furthermore they do not collect all groups of reefs associated species in a representative way^11^.

With increased use of remotely operated vehicles (ROV) in the 1980s, it became possible to study the seafloor and its habitats and organisms directly. Several CWC reefs were discovered during seafloor mapping and pipeline surveys for the Norwegian offshore oil and gas industry^12^. The Sula reef, the Morvin field and the Breisund area are examples of the latter^3^ (and the MAREANO on-line map service: www.mareano.no). However, despite extensive mapping and visual surveys in some geographical areas, knowledge about the distribution of these ecosystems worldwide is still limited.

From present knowledge it seems that geographic location and local conditions, such as prey availability and depth, influence the diet^13^. In some locations CWC appear to mainly be feeding on zooplankton^13–15^. In coastal and oceanic waters in northern Norway copepods are by far the most dominant group within the mesozooplankton, and the most important herbivorous planktotrophic species is the copepod *Calanus finmarchicus*^16^. Wax ester serve as the storage lipid in *C. finmarchicus* and can comprise up to 50% of this copepod’s dry weight^17^. *Calanus finmarchicus* also contains the carotenoid astaxanthin^18^, which is a red pigment often used as a food dye to attain coloration in cultivated salmonids and crustaceans^19^.

In addition to the visual documentation with mobile sensor platforms, an increasing number of fixed underwater observatories (FUO) are equipped with digital cameras. This way visual information with high temporal resolation can be collected over a large time period in order to monitor small areas in CWC ecosystems. The *in-situ* image collections have the potential to provide new insights in ecosystem processes and natural environmental variations on both short (hours and days) and long term (seasons and years). The polyp behaviour of *L. pertusa* has previously been studied to further understand the biology of the coral and to investigate the possibility of using the polyps as an indicator of stress^20–23^. In general, the polyp behaviour reflects feeding patterns, and probably other physiological processes^3,24^. During a petroleum drilling campaign in the Norwegian sea in 2009–2010, the first attempt to measure polyp activity of CWC from a fixed platform *in situ* was made^23^.

Computational support is necessary to efficiently process and interpret the huge amounts of image data gathered both from mobile platforms and fixed ocean observatories. This computational support ranges from data management and development of databases to complex analyses such as image enhancement, segmentation and classification. An additional advantage of computerized image analyses is their objectivity and repeatability. Analyses conducted by humans frequently have low intra-/inter- observer agreement^25^. This problem may be caused by human selective vision and limitation in dividing attention to a variety of simultaneous information^26,27^, such as detecting different organisms and/or their movements over time. Furthermore, detecting small scale changes in object features, such as colour change, is hard for the human brain without a proper scale or colour reference. Several studies have documented that observers have considerable difficulties in recognizing changes in colour or orientation, in particular, when distracting patterns can influence the subjects’ visual attention (see for instance^28,29^).

At present, only a limited number of approaches for computational marine image analysis has been presented. Previous studies have solved different image analysis tasks, such as CWC segmentation in ROV images^30,31^ coral classification^32^, marine resource assessment^33,34^ or megafauna classification^35–37^. Computational methods for image data from stationary observatories have been developed to solve tasks such as object detection/screening^38,39^, monitoring of shrimp distribution in and underneath a *L. pertusa* reef^40^, quantification of colour change in *L. pertusa* over time^41^ and detection and classification of polyp activity in selected parts of a *L. pertusa* reef^42^. Recently, also a short time series study has been published on the gorgonian coral *Paragorgia arborea*, including manual assessment of visual features of the corals^43^. A comprehensive overview for underwater image pre-processing can be found in^44^.

This study represents the next step, where images computationally extracted from a stationary observatory are combined with other sensor data. Image time series and sensor data are obtained from the Lofoten-Vesterålen (LoVe) ocean observatory (love.equinor.com). The aim of the data analyses is to identify and describe, if any, short and long-term dynamics in the behaviour and visual features of *L. pertusa*. The main factors that might influence the behavioural changes are discussed. Short-term dynamics refer here to short periods of hours to days and long-term dynamics are defined as period of months or the total time period analysed, about seven months.

## Material and Field Applications

### The study area

**Figure 1:**
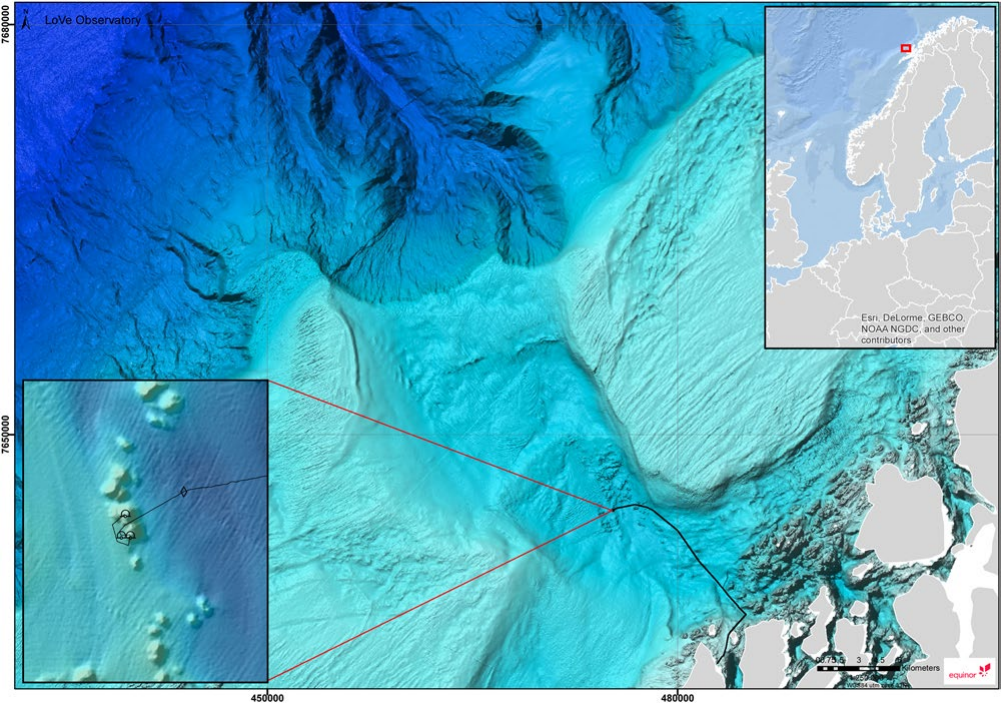
The location of the LoVe observatory. Map generated using Esri ArcGIS 10.2.2 for Desktop. Bathymetry data downloaded from Mareano (http://www.mareano.no/kart/mareano.html#maps/3192) and publicly available. Display of infrastructure data granted by Equinor.

The Lofoten-Vesterålen (LoVe) ocean observatory is located at approximately 250 m depth about 12 km (i.e. ∼20 nautical miles) off the coast of Northern Norway (N 68°54.474′, E 15°23.145′, see Fig. 1). The location is a biological hot spot area in the Norwegian sea, hosting the main spawning area for the North Atlantic cod and has more than 300 *L. pertusa* reefs^45^.

### Equipment and sensors

**Figure 2:**
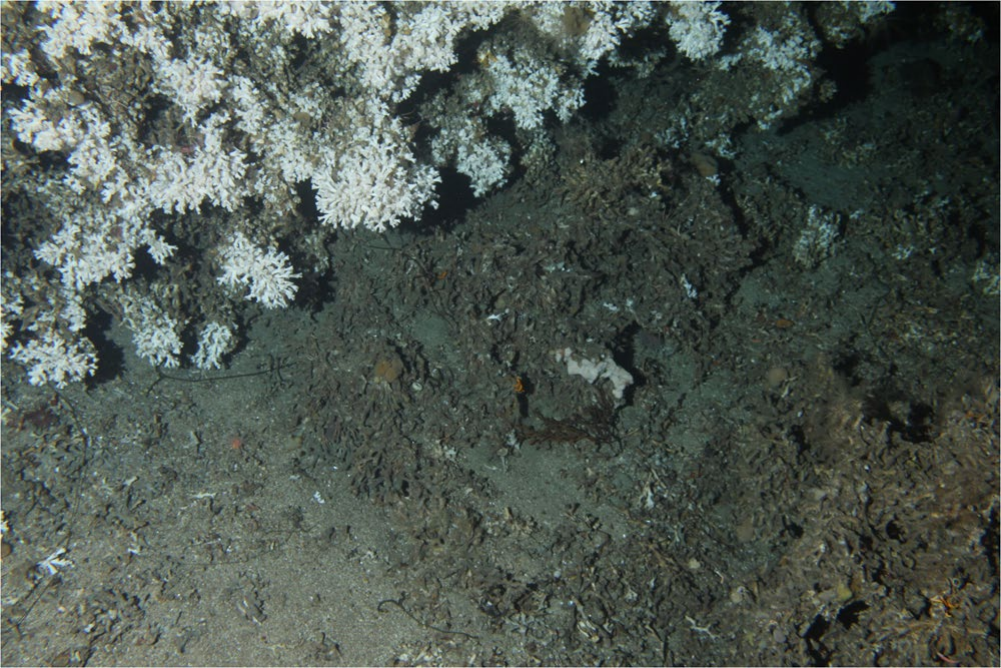
An image recorded at the LoVe observatory the 4^th^ of April 2015 05:59. In the upper left part of the image mostly live CWC can be found, in contrast to the lower right parts where mostly dead coral fragments appear. In between the live coral reef and the coral sand, a coral rubble area were small coral fragments are mixed with sand can be seen.

The ocean observatory was deployed in October 2013. In addition to a waterproof housing (METAS DSF5210) for a digital camera (Canon EOS 550D) and a flash (METAS DSF 4365), the observatory is equipped with a range of sensors (see Table 1 for an overview). In our study, environmental data such as salinity, conductivity, temperature, depth, current speed and direction, and chlorophyll, were correlated with polyp activity. The camera was oriented at an angle of 45° towards a *L. pertusa* reef. The camera covered a visual field of approximately 10 m^2^, including both live parts of the reef and coral rubble as shown in Fig. 2. One image with a resolution of 5184 × 3456 pixels was recorded per hour.

**Table 1:** The temporal resolution of the different sensors *f*_*t*_ used in the multivariate data analysis. Further details can also be found in the online documentation (https://love.statoil.com/Resources/LoVe%20Ocean%20Observatory%20Sensor%20System.pdf).

Feature	Original temporal resolution	Sensor name	Supplier
Temperature ${f}_{t}^{({\rm{T}})}$ [° Celsius]	60 min	AADI 4319A/ADII 4117D	Aanderaa
Depth ${f}_{t}^{({\rm{D}})}$ [m]	60 min	AADI 4319A/ADII 4117D	Aanderaa
Conductivity ${f}_{t}^{({\rm{C}})}$ [mS/cm]	60 min	AADI 4319A	Aanderaa
Salinity ${f}_{t}^{({\rm{S}})}$ [PSU]	60 min	AADI 4319A	Aanderaa
Chlorophyll	60 min	Seapoint Chlorophyll Fluorometer	Seapoint sensors inc.
Current velocity north ${f}_{t}^{({\rm{Vn}}1)}$ [m/s]	10 min	ADCP Aquadopp and ADCP Continental (only the second was used in the final analysis due to the higher time coverage with the analyzed time-period)	Nortek AS
Current velocity east ${f}_{t}^{({\rm{Ve}}1)}$[m/s]	10 min		Nortek AS
Current velocity vertical ${f}_{t}^{({\rm{Vu}}1)}$ [m/s]	10 min		Nortek AS
Current velocity horizontal ${f}_{t}^{({\rm{Vh}}1)}$ [m/s]	10 min		Nortek AS
Coral color *ξ*_*t*_	60 min	Image based	Nortek AS
Polyp activity *γ*_*t*_	60 min	Image based	Nortek AS

Current velocity data were checked for consistency of measurement between the two available ADCPs (Nortek Aquadopp and Nortek Continental) in the overlapping part of their range. ADCP alignment was compared with readings from an independent compass (OASITILT sensor cf. online documentation on the data portal). Environmental sensor data (temperature, salinity/conductivity) were checked for quality using internal consistency checks of the equipment (e.g. comparison of temperature series from the AADI device with that accessory sensors from the ADCPs). Data recorded at the LoVe FUO is publicly available and can be downloaded from http://love.equinor.com.

## Methods

**Figure 3:**
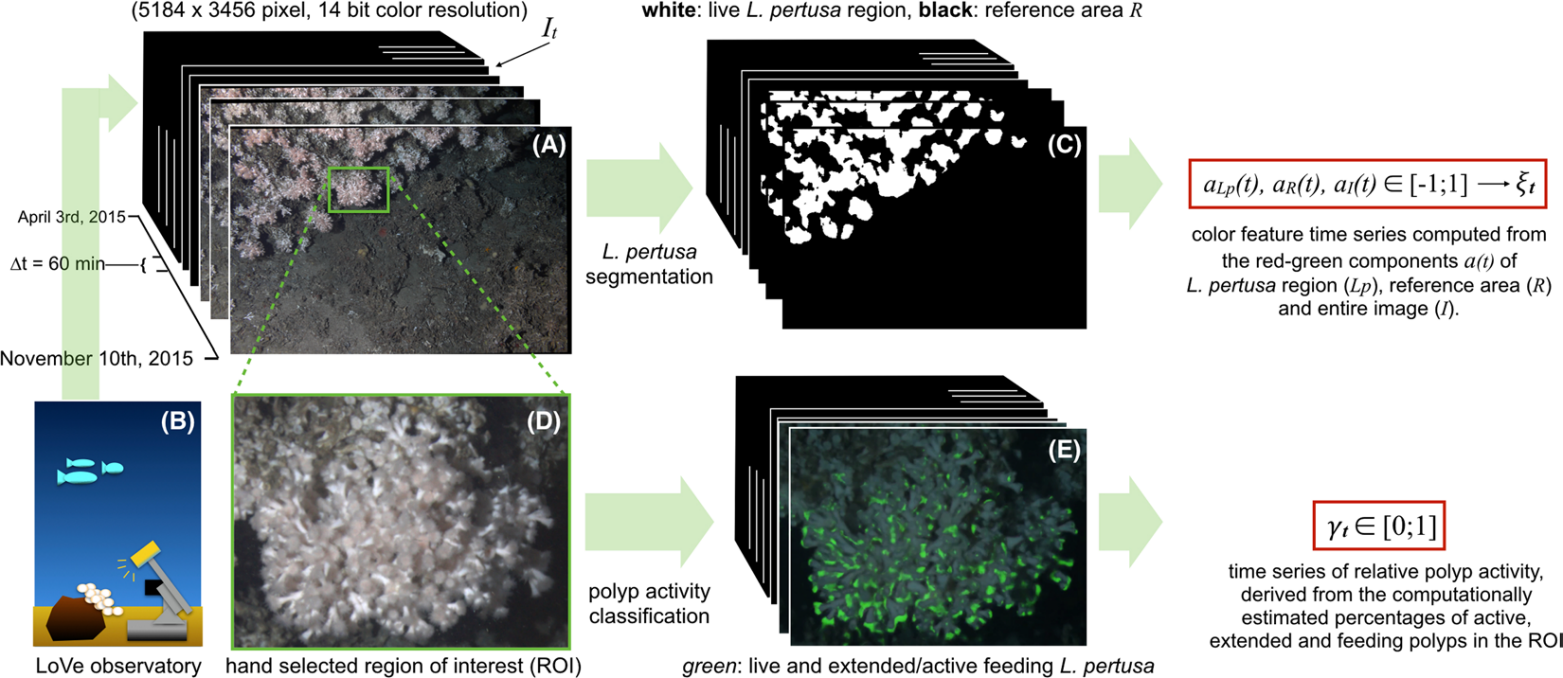
One image (see (**A**)) per hour is uploaded from the LoVe observatory (see schematic graph **(B**), image drawn by author TWN) to the web portal. To compute a time series of colour values (see upper row), the image region of live *L. pertusa* is determined in every single image using a segmentation algorithm (see (**C**) and text for details). Inside the region covered with *L. pertusa*, as well as for the entire image ***I***_***t***_, the average colours are computed in the CIE*Lab* colour space, with the three coordinates *L* (=luminance), *a* (=red-green component) and *b* (=yellow-blue component). The *a*-coordinates of the *L. pertusa* region for all time points were used as time series ***ξ***_***t***_ to describe the red-green component of the considered *L. pertusa* area (see white mask in (**C**)) throughout the observation period. To represent the percentage of active polyps at one time point ***γ***_***t***_ (see lower row) the polyp activity classification algorithm (see the text for details) was applied to each hand selected observation field of view (**D**) to estimate the amount of extended and feeding polyps (green area in (**E**)).

The image sequence from the LoVe observatory which is considered in this study consists of 4862 images {*I*_1_, …, *I*_*N*_} recorded in the time from April 3^rd^ to November 10^th^, 2015. In order to extract coral features time series of interest, we used two machine learning based algorithmic approaches that were applied to each image *I*_*t*_. The first approach was used to compute a coral colour feature *ξ*_*t*_ for each image and time point that describes the colour of the coral. The second approach was used to describe the relative polyp activity *γ*_*t*_ for each image. The approaches are described briefly in the following. A detailed description of all processing steps is also given for both methods in the Supplementary Materials S1 and S2. Additional information can be found in^41,42^. An overview for the computational extraction of both the image-derived time series is shown in Fig. 3. The image analyses have been implemented using the software packages OpenCV (http://openCV.org), NVIDIA DIGITS (http://developer.nvidia.com/digits) and the CAFFE framework (http://caffe.berkeleyvision.org) as well as C++ code developed by the Biodata Mining Group, Bielefeld University. Afterwards we describe the application of linear regression and wavelet coherence analysis that was used to investigate linear relationships between the different time series and their periodicities.

### Coral colour time series computation

Monitoring the corals’ colour change requires automated extraction of colour features from the digital photos that represent the CWC colour at time point *t* (a more detailed description for this method is given in the Supplementary Method S1). First, all images are spatially aligned^46^ and colour transformed (i.e. ${I}_{t}\to {I^{\prime} }_{t}$) with a fixed white balancing. After this pre-processing step, pixel-labelling based segmentation is applied in order to compute a binary mask image ${\hat{M}}^{{\rm{coral}}}$ for the live corals (see Fig. 3 upper middle). The pixel labelling function is constructed using a hierarchical hyperbolic self-organizing map^30,47^, which is trained with Gabor features from a small subset of the images. Then, we computed for each image ${I^{\prime} }_{t}$ the average Cie*Lab* colour values^48^ for the full image (i.e. all pixels)

1 $${\bar{{\boldsymbol{c}}}}_{t}^{{\rm{full}}}=\frac{1}{w\cdot h}\,\cdot \,\sum _{x=1}^{w}\,\sum _{y=1}^{h}\,{I^{\prime} }_{t,(x,y)}$$

and for the live coral colour

2 $${\bar{{\boldsymbol{c}}}}_{t}^{\,\mathrm{full}}=\frac{1}{w\cdot h}\,\cdot \,\sum _{x=1}^{w}\,\sum _{y=1}^{h}{I^{\prime} }_{t,(x,y)}$$

(i.e. only for the pixels inside the mask, see Fig. 3, top right). As the *L*- and the *b*-values did not show any variation of interest we chose the coral colour time series to be the *a*-channel of the live coral colour ${\bar{{\boldsymbol{c}}}}_{t}^{\,\mathrm{coral}}$ as it encodes the complementary red-green component:

3 $${\xi }_{t}={\bar{{\boldsymbol{c}}}}_{t,a}^{\,\mathrm{coral}}.$$

### Polyp activity time series computation

A time series *γ*_*t*_ describing the relative polyp activity (feeding vs. non-feeding) was computed (and a more detailed description can be found in the Supplementary Method S2). To this end, a region of interest (ROI) was chosen that showed CWC with sufficient contrast and in focus (see green frame and lower row in Fig. 3). After pre-processing the images, 13 image ROIs were displayed to human experts (i.e. the authors JJ, IN, PB) to mark positions of active polyps and inactive ones using the annotation software BIIGLE 2.0^49,50^. Based on the three users’ point annotations, a binary mask $\tilde{M}$ inside the ROI was determined that describes the locations of all polyps (i.e. $\tilde{M}$ = 1, for all polyp pixels and = 0, otherwise). In order to train, validate and test a deep neural network, image patches of equal size (46 × 46) were extracted at the positions marked by the human experts, that summed up to 2410 image patches showing polyps. A background class was generated by extracting 100 image patches at random locations in the same ROI but outside the mask $\tilde{M}$. The number of training sample patches was further increased by flipping (augmentation factor: 2x) and rotating (augmentation factor: 12x) the patches and by adding Gaussian noise to them to avoid overfitting (augmentation factor: 2x). Employing these boosting procedures, we achieved a data augmentation by the factor of 48, i.e. an increase of the training data volume by 48-times. Image patches for all categories (active/inactive polyp) are divided into training (70%), validation (20%) and test (10%) patches. Training and validation set were used for training and parameter optimization. The test was left out in in this step so it could be used for a final assessment of the network’s performance for new and “unseen” data. A deep convolutional neural network with a LeNet-5 layout^51^ was trained for classifying image patches to feature an active polyp or an inactive one using the NVIDIA DIGITS software. To finally compute the polyp activity *γ*_*t*_ for one time point, all pixels *(x, y)* inside the mask $\tilde{M}$ are classified by the network to show an element of an active polyp or an inactive one. The network output *C*(*p*_(*x,y*)_) represents the likelihood of the image patch *p*_(*x,y*)_ centered at pixel *(x, y)* showing an active polyp, i.e. *C*(*p*_(*x,y*)_) = 1) or not (i.e. *C*(*p*_(*x,y*)_) = 0) for retracted polyps or other structures. The aggregated number of pixels classified to be “active” in relation to the size of the positive area in $\tilde{M}$ gives the estimated percentage of active polyps for each ROI ${\tilde{I}}_{t}$, referred to as polyp activity *γ*_*t*_ in the rest of this manuscript:

4 $${\gamma }_{t}=\frac{|\,\{{p}_{(x,y)}\in {\mathop{I}\limits^{ \sim }}_{t}|C({p}_{(x,y)})=1\}|}{|\mathop{M}\limits^{ \sim }=1|}.$$

### Multivariate data analysis

In order to investigate functional relations between the introduced image-derived coral colour *ξ*_*t*_ (see eq. 3) and polyp activity *γ*_*t*_ (see eq. 4), and the other physical conditions measured by the other sensors *f*_*t*_ (like temperature ${f}_{t}^{({\rm{T}})}$ or salinity ${f}_{t}^{({\rm{S}})}$), several analyses were conducted. The different sensors of the LoVe observatory have different sampling rates and show varying short episodes of sensor dysfunctions, the latter additionally causing different kinds of temporal delays between measurements of different sensors. Depending on the type of analysis, a mapping and/or interpolation for the different time series was required. In the following we briefly describe the different methods of statistical data analysis we have applied.

To test linear correlations between the image derived time series (*γ*_*t*_ and *ξ*_*t*_) and sensor time series we computed a time series *x*_*t*_ for each sensor *f*_*t*_ that had the same temporal resolution as the image time series. Given an original sensor time series *f*_*t*_ and the original time points $\{t|{f}_{t}\ne \varnothing \}$ of its measurements, we computed the new sensor time series *x*_*t*_ by interpolation

5 $${x}_{t}=\frac{{f}_{{t}^{\ge }}-{f}_{{t}^{\le }}}{{t}^{\ge }-{t}^{\le }}\,\cdot \,(t-{t}^{\le })+{f}_{{t}^{\le }},$$

with

6 $${t}^{\le }=\,{\rm{\max }}\{{t}^{\ast }|{t}^{\ast }\le t\wedge {t}^{\ast }\in \{t|{f}_{t}\ne \varnothing \}\}$$

and

7 $${t}^{\ge }=\,{\rm{\min }}\{{t}^{\ast }|{t}^{\ast }\ge t\wedge {t}^{\ast }\in \{t|{f}_{t}\ne \varnothing \}\}.$$

Pairs of time-series were plotted in scatterplots to identify potential relationships between data sets. Linear correlation was tested between polyp activity*, γ*_*t*_ and time series *x*_*t*_. Daily averages (${\bar{\gamma }}_{t},\,{\bar{x}}_{t}$) of *γ*_*t*_ and *x*_*t*_ were computed and tested for pair-wise correlation. For the latter, no linear interpolation was applied, ignoring days where no data was recorded either for *γ*_*t*_ and/or *x*_*t*_.

Wavelet-based analysis methods were performed 1) to identify dominant frequencies/scales that contribute to the overall variance in individual time series, and 2) to assess covariation between the image-derived time series and the environmental variables measured by the other sensors. In contrast to a Fourier transformation-based analysis, which discards the time dimension when data are transformed to frequency space, the wavelet transformation retains time and frequency/scale information, thus facilitating identification of patterns in data that have limited temporal extent^52^. Dominant frequencies/scales in individual time series were identified in the bias-corrected wavelet power spectrum^53,54^. The default Chi^2^-test from the R function was used to test for significance of variance contributions at the 95% significance level. Scale- and time dependent covariation between two time series was investigated with a wavelet coherence analysis^55^. Wavelet coherence is the wavelet transformation analogue of a cross-correlation analysis or a Fourier coherence analysis. It is calculated from the wavelet cross-spectrum of two series by dividing it by the power spectra of the individual time series^56^. It is reported as a squared value between 0 and 1, similar in interpretation to a squared correlation. Significance of the wavelet variance and coherence was tested by Monte Carlo randomization tests^54^.

Since the methodology behind the biwavelet package assumes equidistant time points for the analysis of a time series (and identical temporal resolution for a pairwise analysis) the time series must be transformed accordingly. Estimated polyp activity *γ*_*t*_, coral color ${\xi }_{t}$ with the original time points of imaging $\{t|{\gamma }_{t}\ne \varnothing \}$ were therefore converted to have equidistant time points ($t=1\,{\rm{hour}}$) by linear interpolation:

8 $$\gamma {\text{'}}_{t}=\frac{{\gamma }_{{t}^{\ge }}-{\gamma }_{{t}^{\le }}}{{t}^{\ge }-{t}^{\le }}\cdot (t-{t}^{\le })+\,{\gamma }_{{t}^{\le }}$$

with

9 $${t}^{\le }=\,\max \{{t}^{\ast }|{t}^{\ast }\le t\wedge {t}^{\ast }\in \{t|{\gamma }_{t}\ne \varnothing \}\}$$

and

10 $${t}^{\ge }=\,\min \{{t}^{\ast }|{t}^{\ast }\ge t\,\wedge {t}^{\ast }\in \{t|{\gamma }_{t}\ne \varnothing \}\}.$$

To allow a pairwise analysis of image-derived time series with other sensor data *f*_*t*_ was mapped (${f}_{t}\to {x^{\prime} }_{t}$) to interpolate measurements for those time points of the set $\{t|{\gamma ^{\prime} }_{t}\ne \varnothing \}$. The wavelet transformation was applied to each individual time series with a Morlet mother wavelet function, followed by pairwise coherence wavelet analysis between *γ*_*t*_ and all *x*_*t*_. The analyses were conducted in the R Statistical software (R Core Team, 2016) using the addon package “biwavelet”^57^.

## Results

To assess the accuracy in polyp activity, several processing approaches were evaluated and compared. A comparison of the human expert annotations showed an inter-observer agreement of 0.86 and an intra-observer agreement of 0.89. The classification accuracy of the trained network was 0.98 for the training data and 0.96 for the validation and test data. The accuracy of the relative polyp activity *γ*_*t*_ was assessed for the 13 images, that were annotated by the human experts (more details are given in the Supplementary S2). From the manual annotations, another value for relative polyp activity was computed and compared to *γ*_*t*_. A spearman’s rank correlation of the two measurements was calculated to be 0.98.

**Figure 4:**
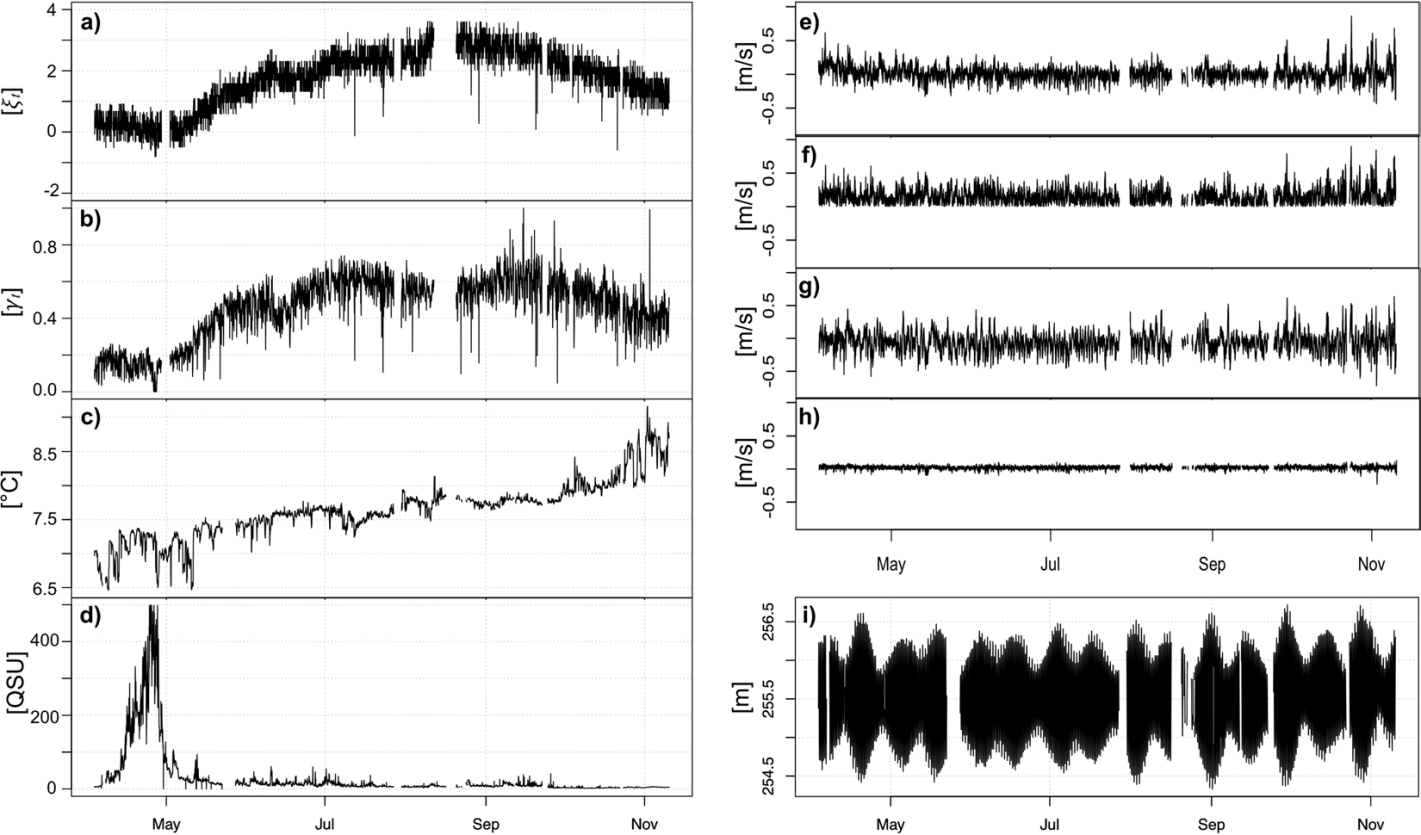
Measurements of selected sensors are plotted together with estimated polyp activity ***γ***_***t***_ and coral colour ***ξ***_***t***_ (*a*-coordinate of the coral colour ${\bar{{\boldsymbol{c}}}}_{{\boldsymbol{t}}}^{\,\mathrm{coral}}$). Plot (**a**) shows the development of the colour value ***ξ***_***t***_ that describes the redness of the color structures. An increase of ***ξ***_***t***_ reflects a change of the colour into a more reddish hue. In mid of May the colour starts to change into red. Plot (**b**) shows the corresponding relative polyp activity at each time point ***γ***_***t***_. Gaps in the series originate in camera malfunctions. Plot (**c**) shows the temperature and (**d**) the measured concentration of chlorophyll with a high peak at the end of April. In (**e–h**) the four plots of the current velocity measurements are shown (with **e**) Ve1, (**f**) Vh1, (**g**) Vn1, (**h**) Vu1). Plot (**i**) shows the distance from the bottom to the sea surface.

An overview of all data attributes and temporal resolutions is given in Table 1. Raw data are displayed in Fig. 4a–i. As can be seen in the plots, the time series show some gaps due to sensor problems and/or break downs of the communication with the ocean observatory.

Nevertheless, the time series of polyp activity, coral colour as well as temperature and conductivity (data not shown) show long-term trends and patterns with marked changes starting in May.

The colour value (***ξ***_***t***_), describing the redness of the polyp tissue, changes gradually towards more reddish hue from mid of May to August (Fig. 4a). The onset of this change seems to follow a marked peak in the chlorophyll concentration at the end of April (Fig. 4d). The current velocity did not display any clear seasonality patterns (Fig. 4e–h). The variation in depth reflects the tidal variation (Fig. 4i).

**Figure 5:**
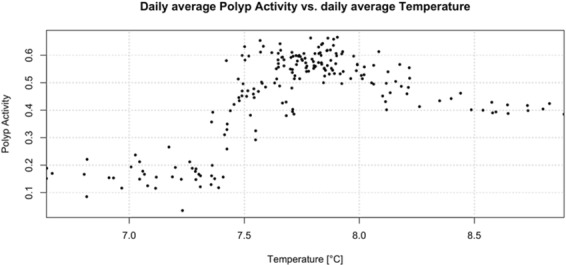
The daily averages for temperature ($\overline{{x}_{t}^{({\rm{T}})}}$) are plotted against daily averages for the polyp activity ($\overline{{\gamma }_{t}}$). The estimated pearson correlation coefficient between both is 0.54.

Polyp activity *γ*_*t*_ increased rapidly between a bottom water temperature 7.4 and 7.5 °C and reached a maximum between 7.5 and 8.0 °C (Fig. 5). At higher bottom water temperatures polyp activity decreased slightly to remain more or less constant beyond temperatures of 8.3 °C (Fig. 5). Pearson correlation coefficients (Supplementary Table S3) for pairwise correlation of *γ*_*t*_ and ${x}_{t}^{(C)}$, ${x}_{t}^{(T)}$, ${x}_{t}^{(S)}$ were not high. The estimated coefficients for the daily average were higher but still below 0.8. Looking at the correlation between polyp activity and current, $r({\gamma }_{t},\,{x}_{t}^{({\rm{Vu}}1)})$ and $r(\overline{{\gamma }_{{t}^{I}}},\overline{{x}_{t}^{({\rm{Vu}}1)}})$, the values indicated no linear dependency.

**Figure 6:**
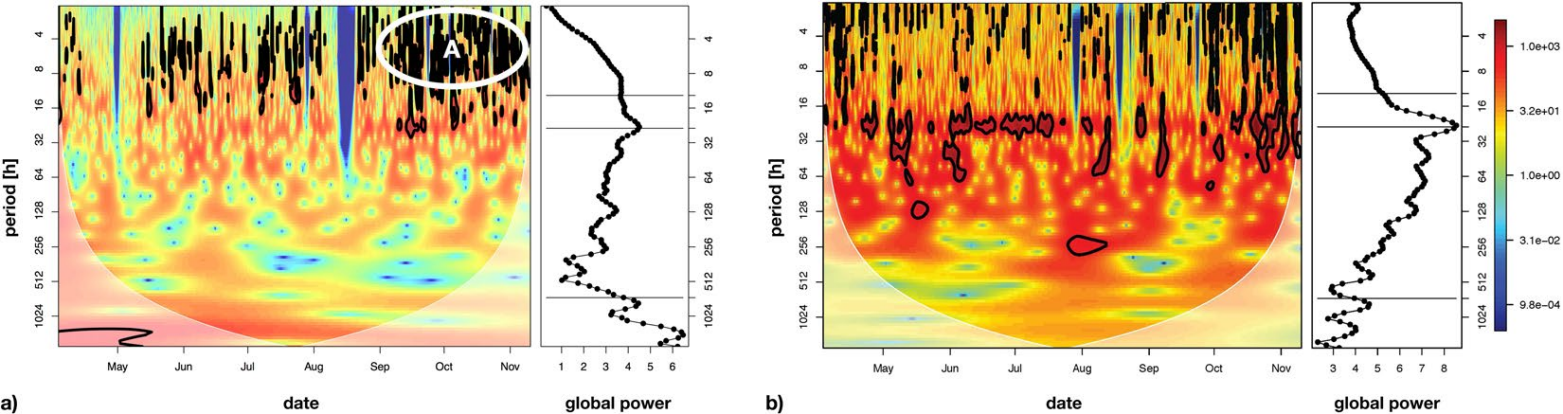
The wavelet power spectrum (see left panels in the subplots) of the polyp activity (${\gamma ^{\prime} }_{t}$) (see **a**)) northward bottom current velocity (${x}_{t}^{(Vn1)}$) (see **b**)) and the corresponding scale-averaged global power spectra (right panels in the subplots). Significant contributions to the time series’ variance (Chi^2^-test, 95% significance level) are indicated by the black contours. The horizontal lines in the global wavelet spectra mark the tidal scale (12.4 h, daily (24 h) and lunar (~707 h) scales.

The periodicity of the different time series was analysed using wavelet transformations. Linear interpolation was used to compute time series with equidistant time points from *γ*_*t*_ and *f*_*t*_ (see equation (8)). First, single time series were analysed with respect to periodic patterns, starting with the polyp activity *γ*_*t*_. The result of this analysis is visualized in Fig. 6a.

The wavelet spectrum of the polyp activity *γ*_*t*_ exhibited only statistically significant variance contributions at scales below 16 hours (black contours in Fig. 6a (left panel), plots without the black contours are shown in the Supplementary S4). These statistically significant regions occurred throughout summer, but particularly from September to November (region indicated with “A” in Fig. 6a), left panel). Although no significant variance contribution in poly activity *γ*_*t*_ was detected at the 24-hour scale band in the wavelet variance plot (Fig. 6a, left panel), the corresponding global wavelet variance spectrum (right panel in Fig. 6a), averaged by scale, exhibited a distinct peak at the 24-hour scale, indicating that polyps showed some increased variation in feeding activity with a 24-hour scale. Additional variance peaks were also found at longer scales, including that of the lunar cycle (approx. 700 h = ~29 days).

The wavelet spectral plot (Fig. 6b (left panel), a plot without black contours is shown in the Supplementary S4) and global averaged wavelet spectrum (Fig. 6b, right panel) of the northward component of the bottom current velocity (Vn1) showed a significant and dominant contribution of the 24-hour scale to the overall variance, indicative of a tidal dominance in the current velocity. A bit surprisingly, no spectral peaks were found at the 12.5 h scale, which may be due to interactions of the tidal wave with the basin morphology. Some significant contributions of smaller time scales were found as well but were mainly restricted to the beginning and end of the time series.

Similar analyses of the wavelet variance demonstrated a strong contribution of the lunar phase to the overall variance in the eastward current velocity component and a dominant tidal (12.5 h) contribution to the water depth (data not shown).

**Figure 7:**
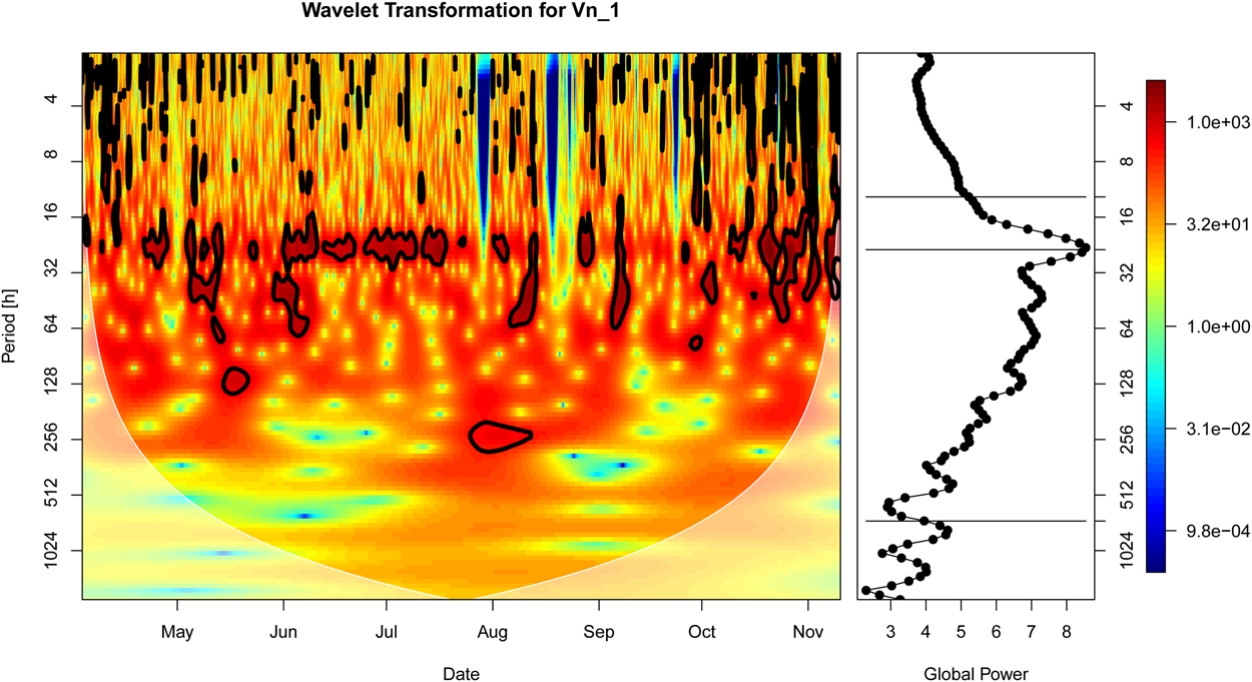
Left panel: Squared wavelet coherence (colour) and phase (arrows) between polyp activity $({{\boldsymbol{\gamma }}{\boldsymbol{^{\prime} }}}_{{\boldsymbol{t}}})$ and the northward component of the bottom current velocity $({{\boldsymbol{x}}}_{{\boldsymbol{t}}}^{{({\boldsymbol{Vn}}1)}^{{\boldsymbol{\text{'}}}}})$. A value of 1 would indicate a perfect coherence, while a value of 0 would indicate no coherence. Significant coherence is indicated by the black contour lines (Monte Carlo test, 95% significance level). Right panel: The corresponding scale-averaged coherence over the entire measurement period.

Significant coherence (covariation) between the polyp activity and the northward current velocity was found at various scales up to 256 hours (~10 days), throughout the entire measurement period (Fig. 7 (left panel) and Supplementary Fig. S5). At the 24-hour scale the two signals tended to be in phase (arrows predominantly pointing to the right in Fig. 7 left panel), whereas they tended to be in counter-phase at larger scales (arrows predominantly pointing to the left).

In the Supplementary Fig. S6 we show a plot of polyp activity and current velocity Vn1 for a selected interval of 4 weeks to illustrate coherences (see Supplementary Fig. S3).

## Discussion

Our results show that the polyp behavior of the monitored *L. pertusa* display different, alternating temporal patterns, or periodicities. The analyses indicate that the main factors influencing the polyp behavior and color varies both with respect to the internal relationship between the various factors measured and with time. Behavioral periodicity has been observed in many warm water corals (see references in^58^) but such patterns have not been previously documented in CWC *in situ*.

### Long term dynamics

In general, the long-term (7 months) trend of polyp activity and coral color (Fig. 4a,b) follow a similar pattern with a low level from April to May, followed by a gradual increase from May to July. The onset of the increase follows an early sharp peak in chlorophyll content (Fig. 4d). This peak marks the spring bloom of phytoplankton, which is the basis for the growth of the zooplankton communities, dominated by calanoid copepods. The long-term change in the colour in the *L. pertusa* reef (Fig. 4a) was first published in 2016^38,41^ and is further supported by our study. We suggest that the colour changes documented in *L. pertusa* between April and November, is a result of increased feeding on calanoid copepods containing the red pigment astaxanthin. According to^13^ and^15^, copepods and other planktonic animals within reach of the reefs during summer constitute the main food sources for *L. pertusa* together with phytodetritus^59,60^. The increased polyp activity is likely to be related to feeding activity, and as a high food uptake continues throughout the summer and early fall, the pigments from the food probably accumulate in the coral tissue. Changes in polyp activity related to feeding activity has previously been documented in laboratory experiments^61,62^. The calanoids show both seasonal and diurnal vertical migration patterns. During winter, the populations enters diapause in deep and cold water. Patches of high abundance of overwintering *C. finmarchicus* in the Lofoten Basin, just west of our study site, has been documented at 550–800 meters^63^. In mid-late winter, the population ascends to surface waters where it matures and spawns (see for example^64^). During its life cycle *C. finmarcicus* goes through six Nauplius and five Copepodite stages (CI-CV). The maximum abundance of the first generation CI often occur during or right after the peak of the phytoplankton bloom (reviewed in^65^). In June and July, the first generation of CIV and CV starts to migrate into the deep for diapause and a second generation starts their migration in August and September^16,63,66,67^.

In addition to the seasonal migration, the calanoid perform diurnal vertical migration (DVM). This is a strategy to avoid visual predators (e.g. fish)^68–70^. Copepods reside deeper in the water column during the day than at evening and night time, when they migrate towards the sea surface to graze on phytoplankton (e.g.^66^).

With the maturation of the calanoids, the depth of the migration increases towards the end of the summer^16,66,69^ before it is followed by the migration to diapause. The CWC habitats in Norway are relatively shallow^71^ and it is therefore likely that the seasonal migration, in combination with the DVM, accommodate the CWC with high quality food during summer and autumn.

Our suggestion that copepods are causing the colour change in *L. pertusa* is further supported by^16^, showing that the storage lipid signature of *L. pertusa* contained a higher level of calanoid copepod lipid biomarkers at the shallow Mingulay reef (130 m) than the deeper reefs at Rockall (900 m) and New England (1200–1300 m). Additionally, the Mingulay corals contained the highest number of wax esters of the sites, which is the storage lipid of the copepod *C. finmarchicus*^17^.

Temperature (Fig. 4c) also shows long-term seasonal changes, with a steady increase from April/May until end of the measuring period in mid-November. Between May and September, there is a positive correlation (r^2^ = 0.54) between polyp activity and temperature. Such a correlation has also been observed in laboratory experiment^72^. However, from September until mid-November, the temperature continues to increase while the polyp activity decreases. *L. pertusa* occur within a rather wide temperature range worldwide, from 4 to 14 °C, and has a high tolerance to an unpredictable temperature regime^73^. This may suggest that food availability influence polyp activity to a higher degree than temperature. But given duration of the measurements and the lack of verification of food available in the data, there are currently not enough data available to conclude on how the temperature or a combination of temperature and food influences the polyp activity of *L. pertusa*. Analyses on longer time series in the future will determine whether the correlation with temperature and other drivers at the seasonal scale is of ecological significance or just an artefact of the cyclicity in two auto-correlating time series with a limited temporal extent.

### Short term patterns

The short term (hours and days) dynamics (i.e. short periodicities) of the different sensors seem to be more complex than the long-term (months) changes controlled by factors varying both diurnally (Figs 6 and 7) and with the lunar phase (tidally).

The wavelet coherent study for current speed and polyp activity showed a match for 24 hours and an approximate match for 33 days. The results from the coherence analysis suggest opposite responses of polyp feeding activity to tidal currents relative to residual currents (larger than 24-hour). However, the coherence at the 24-hour scale does not necessarily have to originate from tidal variability. Daily vertical migration of prey species could also induce variability of polyp feeding activity at a 24-hour scale. From August onwards significant coherence was also detected at the lunar scale. Signals were roughly in counter-phase at this scale.

Even though no long-term pattern was documented for current speed and direction in the present study, it is documented that food transport to corals is directly linked to current speed and direction^74^. Laboratory studies have shown that slow current speeds, with velocities less than 7 cm/sec, are optimal for feeding in CWC^61,62^. At higher flow velocity fewer polyps are active, likely due to greater drag on the tentacles and lower probability of food capture^61,62^. The authors of^62^ also describe the variety of food capture efficiency with food source in addition to water flow. Furthermore, also a field study^13^ has indicated changes in the proportion of polyps in different expansion states caused by natural stress from water motion around the polyps and/or by different water quality or food content related to changes in current speed and direction.

The tidal wavelength of 12.25 hours is directly linked to variation in current speed and direction. Because of a tidal periodicity of 12.25 hour, high and low tide does not occur at the same time every day. This implies that there are days within this cycle when the currents are disadvantageous while the zooplankton is present around the corals, and vice versa. During a tidal cycle, highest current velocities occur twice (6.12 hours apart). A combination of wave patterns can feature interferences and this may explain why the corals’ polyp behaviour seems to swap between different periodicities (Fig. 6a).

Our findings demonstrate the importance of using a sampling frequency relevant for capturing dynamic patterns at the scale they occur. Which time scale is relevant for sampling of biological and environmental data depends on the aim of the study.

### Conclusions and future perspectives

The high temporal resolution and coverage of the data measured at the LoVe ocean observatory opens up for a new insight, and jet unknown opportunities for understanding natural variations and the dynamic in CWC reefs. These first results reveal that various parameters influence *L. pertusa* at different time scales, and that the various periodicities also influence each other.

Understanding the natural variations and dynamics influencing key species or ecosystems is essential to interpret environmental monitoring data.

To gain more insight and knowledge about these interactions there is first and foremost a need to gather more experience and better statistics for both long- and short-term changes. Furthermore, to generalize the present findings, the methodology must be further developed for applications in broader areas. The results also show that to reduce the uncertainty in our analysis there is a need of technology development of sensors that can measure and specify food availability with high temporal frequency and coverage. The documentation of *L. pertusa* changing color through the year is one example of new knowledge that is a result of interdisciplinary collaboration on the LoVe data.

In a broader perspective, the anticipation of *L. pertusa* feeding on *C. finmarcicus* causing this color change indicates a strong predator-prey interaction between the two species. We can therefore expect that fluctuations in zooplankton also could influence *L. pertusa*’s general health and reproductive cycles. With more than 10.000 *L. pertusa* reefs documented in Norwegian waters this new knowledge should also be considered included in CWC ecosystem modelling. As indicated in this study *L. pertusa* might be an important predator that at present is missing in the modelling. Ultimately, this new insight could also have implications for understanding details in food web dynamics involving commercial important fish stocks grazing on copepods, such as herring.

## Supplementary information

Supplementary information

Data Matrix

## Data Availability

All Images and sensor data used in this work were made available via the Lofoten-Vesterålen Ocean Observatory web portal (love.statoil.com). The single time series are also provided with this submission (data_matrix.zip).

## References

[1] Buhl-Mortensen P (2017). Coral Reefs in the Southern Barents Sea: Habitat Description and the Effects of Bottom Fishing. Marine Biology Research.

[2] Costello, M. J. *et al*. Role of cold-water *Lophelia pertusa* coral reefs as fish habitat in the NE Atlantic. Cold-Water Corals and Ecosystems. *Springer Berlin Heidelberg*, 771–805 (2005).

[3] Mortensen P, Hovland T, Fosså JH, Furevik DM (2001). Distribution, abundance and size of *Lophelia pertusa* coral reefs in mid-Norway in relation to seabed characteristics. Journal of the Marine Biological Association of the UK.

[4] Wheeler AJ (2007). Morphology and environment of cold-water coral carbonate mounds on the NW european margin. International Journal of Earth Sciences.

[5] Van Oevelen D (2009). The cold-water coral community as a hot spot for carbon cycling on continentalmargins: A food-web analysis from Rockall Bank (northeast Atlantic). Limnology and Oceanography.

[6] Wagner H, Purser A, Thomsen L, Jesus CC, Lundälv T (2011). Particulate organic matter fluxes and hydrodynamics at the Tisler cold-water coral reef. Journal of Marine Systems.

[7] White M (2012). Cold-water coral ecosystem (Tisler Reef, Norwegian Shelf) may be a hotspot for carbon cycling. Mar. Ecol. Progr. Ser..

[8] Dons, C. Norges Korallrev. Det Kongelige Norske Videnskabers Selskabs Forhandlinger **16**, 37–82 (1944).

[9] Burdon-Jones C, Tambs-Lyche H (1960). Observations on the fauna of the North Brattholmen stone-coral reef near Bergen. Årbok for Universitetet i Bergen. Mat.-naturv. Serie..

[10] Jensen A, Frederiksen R (1992). The fauna associated with the bank-forming deepwater coral *Lophelia pertusa* (Scleractinaria) on the Faroe shelf. Sarsia.

[11] Mortensen, P. & Fosså, J. H. Species Diversity and Spatial Distribution of Invertebrates on Deep–water Lophelia Reefs in Norway. Proceedings of 10th International Coral Reef Symposium 1849–68 (2006).

[12] Hovland, M., Farestveit, R. & Buhl-Mortensen, P. Large cold-water coral reefs off mid-norway - a problem for pipe-laying? Oceanology International. Brighton, UK (1994).

[13] Dodds LA, Black KD, Orr H, Roberts JM (2009). Lipid biomarkers reveal geographicaldifferences in food supply to the cold-water coral Lophelia pertusa (Scleractinia). Mar. Ecol. Progr. Ser..

[14] Kiriakoulakis, K. *et al*. Lipids and nitrogen isotopes of two deep-water corals from the north-east atlantic: Initial results and implications for their nutrition. *Cold-Water Corals and Ecosystems*. 715–29 (2005).

[15] Carlier A (2009). Trophic relationships in a deep mediterranean cold-water coral bank (Santa Maria di Leuca, Ionian Sea). Mar. Ecol. Progr. Ser..

[16] Johanson AN (1999). Physical and biological factors influencing the seasonal variation in distribution of zooplankton across the shelf at Nordvestbanken, northern Norway, 1994. Sarsia..

[17] Sargent JR, Gatten RR, Henderso RJ (1981). Marine wax esters. Pure and Applied Chemistry..

[18] Foss P, Renstrom B, Liaaenjensen S (1987). Natural occurrence of enantiomeric and meso astaxanthin 7-star-crustaceans including zooplankton. Comparative Biochemistry and Physiology, Part B: Biochemistry and Molecular Biology..

[19] Higuera-Ciapara I, Felix-Valenzuela L, Goycoolea FM (2006). Astaxanthin: A review of its chemistry and applications. Critical Reviews in Food Science and Nutrition..

[20] Shelton GAB (1980). Lophelia pertusa (L.): Electrical conduction and behaviour in a deep-water coral. Journal of the Marine Biological Association of the UK.

[21] Serigstad, B., Mangor-Jensen, A. & Mortensen, P. B. Effects of oil on marine deep-sea organisms. Institute of Marine Research, Report No 2b/2001. 38 pages. (in Norwegian) (2001).

[22] Roberts JM, Anderson RM (2002). A new laboratory method for monitoring deep-water coral polyp behaviour. Hydrobiologia.

[23] Buhl-Mortensen P, Tenningen E, Tysseland ABS (2015). Effects of water flow and drilling waste exposure on polyp behaviour in *Lophelia pertusa*. Marine Biology Research.

[24] Larsson AI, van Oevelen D, Purser A, Thomsen L (2013). Tolerance to long-term exposure of suspended benthic sediments and drill cuttings in the cold-water coral *Lophelia pertusa*. Marine Pollution Bulletin.

[25] Durden JM (2016). Comparison of image Annotation Data Generated by Multiple Investigators for Benthic Ecology. Mar. Ecol. Progr. Ser..

[26] Carrasco, M., Ling, S. & Read, S. (2004) Attention alters appearance. *Nat Neuroscience*. **7**(3), 10.1038/nn1194 (2004). 10.1038/nn1194 PMC3882082 14966522

[27] Chun MM, Golomb JD, Turk-Browne NB (2011). A taxonomy of external and internal attention. Ann. Rev. Psychol..

[28] Rensink RA, O’Regan JK, Clark JJ (2000). On the failure to detect changes in scenes across brief interruptions. Vis. Cogn..

[29] Wolfe JM, Reinecke A, Brawn P (2006). Why Don’t we see changes? The role of attentional bottlenecks and limited visual memory. Vis. Cogn..

[30] Purser A, Bergmann M, Lundälv T, Ontrup J, Nattkemper TW (2009). Use of machine-learning algorithms for the automated detection of cold-water coral habitats: A Pilot Study. Mar. Ecol. Progr. Ser..

[31] Tusa, E. *et al*. Implementation of a fast coral detector using a supervised machine learning and gabor wavelet feature descriptors. Proc. of Sensor Systems for a Changing Ocean (SSCO), IEEE. 1–6 (2014).

[32] Beijbom O (2015). Towards automated annotation of benthic survey images: Variability of human experts and operational modes of automation. PLoS One.

[33] Schoening T, Kuhn T, Jones DOB, Simon-Lledo E, Nattkemper TW (2016). Fully automated image segmentation for benthic resource assessment of poly-metallic nodules. Methods in Oceanography..

[34] Schoening, T., Jones, D. & Greinert, J. Compact-morphology-based poly-metallic nodule delineation. *Scientific Reports*, **7**(13338), 10.1038/s41598-017-13335-x (2017). 10.1038/s41598-017-13335-x PMC5645364 29042585

[35] Schoening T (2012). Semi-automated image analysis for the assessment of megafaunal densities at the arctic deep-sea observatory HAUSGARTEN. PloS One.

[36] Langenkämper D. & Nattkemper T.W.COATL - a learning architecture for online real-time detection and classification assistance for environmental data. Proc. 23rd International Conference on Pattern Recognition (ICPR). Cancun, Mexico, 597–602 (2016).

[37] Zurowietz M, Langenkämper D, Hosking B, Ruhl HA, Nattkemper TW (2018). MAIA—A machine learning assisted image annotation method for environmental monitoring and exploration. PLoS One.

[38] Möller, T., Nilssen, I. & Nattkemper, T. W. Change detection in marine observatory image streams using bi-domain feature clustering. Proc. 23rd International Conference on Pattern Recognition (ICPR), pp. 793–98, Cancun, Mexico (2016).

[39] Möller, T., Nilssen., I. & Nattkemper, T. W. Active learning for the classification of species in underwater images from a fixed observatory, Proc. of IEEE International Conference on Computer Vision Workshops (ICCVW), Venice, 2891–7 (2017).

[40] Osterloff J, Nilssen I, Nattkemper TW (2016). A computer vision approach for monitoring the spatial and temporal shrimp distribution at the LoVe observatory. Methods in Oceanography.

[41] Osterloff, J., Nilssen, I., & Nattkemper, T. W. Computational coral feature monitoring for the fixed underwater observatory LoVe. Proc. of OCEANS 2016 MTS/IEEE Monterey, 1–5 (2016).

[42] Osterloff, J., Nilssen, I., Jarnegren, J., Buhl-Mortensen, P. & Nattkemper, T. W. Polyp activity estimation and monitoring for cold water corals with a deep learning approach. Proceedings - 2nd Workshop on Computer Vision for Analysis of Underwater Imagery (CVAUI), 1–6 (2016).

[43] Johanson AN, Flögel S, Dullo WC, Linke P, Hasselbring W (2017). Modeling polyp activity of *Paragorgia arborea* using supervised learning. Ecological Informatics.

[44] Schettini, R. & Corchs, S. Underwater image processing: State of the art of restoration and image enhancement methods. *EURASIP Journal on Advances in Signal Processing*, 1–15 (2010).

[45] Bøe, R. *et al*. Cold-water coral reefs in the Hola glacial trough off Vesterålen, North Norway. In Dowdeswell, J. A., Canals, M., Jakobsson, M., Todd, B. J., Dowdeswell, E. K. & Hogan, K. A. (eds) Atlas of Submarine Glacial Landforms: Modern, Quaternary and Ancient. *Geological Society, London, Memoirs*, **46**, 309–10, 10.1144/M46.8 (2016).

[46] Evangelidis GD, Psarakis EZ (2008). Parametric image alignment using enhanced correlation coefficient maximization. IEEE Transactions on Pattern Analysis and Machine Intelligence.

[47] Osterloff J, Nilssen I, Eide I, Nattkemper TW (2016). Computational visual stress level analysis of calcareous algae exposed to sedimentation. PLoS One.

[48] Gevers, T., Gijsenij, A., van de Weijer, J. & Geusebroek, J. M. Color in Computer Vision: Fundamentals and Applications. Wiley, ISBN 978-0470890844 (2012).

[49] Langenkämper, D., Zurowietz, M., Schoening, T. & Nattkemper, T. W. BIIGLE 2.0 - Browsing and annotating large marine image collections. *Frontiers in Marine Science* **4** (2017).

[50] Schoening, T., Osterloff, J. & Nattkemper, T. W. RecoMIA—Recommendations for marine image annotation: Lessons learned and future directions. *Frontiers in Marine Science* **3** (2016).

[51] LeCun, Y., Bottou, L., Bengio, Y. & Haffner, B. Gradient-based learning applied to document recognition. *Proceedings of the IEEE* **86**(11), 2278–324, 10.1109/5.726791. arXiv: 1102.0183 (1998).

[52] Shumway, R. H. & Stoffer, D. S. Time Series Analysis and its Applications, Springer, ISBN 978-3-319-52452-8 (2017).

[53] Liu Y, San Liang X, Weisberg RH (2007). Rectification of the bias in the wavelet power spectrum. Journal of Atmospheric and Oceanic Technology.

[54] Veleda D, Montagne R, Araujo M (2012). Cross-wavelet bias corrected by normalizing scales. Journal of Atmospheric and Oceanic Technology.

[55] Grinsted A, Moore JC, Jevrejeva. S (2004). Application of the cross wavelet transform and wavelet coherence to geophysical time series. Nonlinear Processes in Geophysics.

[56] Torrence C, Compo GP (1998). A practical guide to wavelet analysis. Bulletin of the American Meteorol. Soc..

[57] Gouhier, T. C. & Grinsted, A. & Simko, V. R Package ‘biwavelet’: Conduct Univariate and Bivariate Wavelet Analyses, https://github.com/tgouhier/biwavelet (2017).

[58] Hoadley, K. D., Szmant, A. M. & Pyott, S. J. Circadian clock gene expression in the coral *Favia fragum* over diel and lunar reproductive cycles. *PLoS One*. **6** (2011) 10.1371/journal.pone.0019755 PMC3089635 21573070

[59] Kiriakoulakis K, Bett BJ, White M, Wolff GA (2004). Organic biogeochemistry of the Darwin Mounds, a deep-water coral ecosystem, of the NE Atlantic. Deep-Sea Res. I.

[60] Duineveld GCA, Lavaleye MSS, Berghuis EM (2004). Particle flux and food supply to a seamount cold-water coral community (Galicia Bank, NW Spain). Mar. Ecol. Progr. Ser..

[61] Purser A, Larsson AI, Thomsen L, van Oevelen D (2010). The influence of flow velocity and food concentration on *Lophelia Pertusa* (Scleractinia) Zooplankton Capture Rates. Journal of Experimental Marine Biology and Ecology.

[62] Orejas C (2016). The effect of flow speed and food size on the capture efficiency and feeding behaviour of the cold-water coral Lophelia pertusa. Journal of Experimental Marine Biology and Ecology.

[63] Halvorsen E, Tande KS, Edvardsen A, Slagstad D, Pedersen OP (2003). Habitat selection of overwintering Calanus finmarchicus in the NE Norwegian Sea and shelf waters off Northern Norway in 2000–02. Fisheries Oceanography.

[64] Melle, W., Ellertsen, B. & Skjoldal, H. R. Zooplankton: the link to higher trophic levels. In: Skjoldal, H. R. (ed.) *The Norwegian Sea ecosystem*. Tapir Academic Press, Trondheim, 137–202 (2004).

[65] Melle W (2014). The North Atlantic ocean as habitat for Calanus finmarchicus: Environmental factors and life history traits. Progress in Oceanography.

[66] Falkenhaug T, Tande KS, Semenova T (1997). Spatio-temporal patterns in the copepod community in Malangen, Northern Norway. Journal of Plankton Research..

[67] Pedersen PO, Tande KS, Slagstad D (2001). A model study of demography and spatial distribution of Calanus finmarchicus at the Norwegian coast. Deep-sea. Research II.

[68] Hays GC, Kennedy H, Frost BW (2001). Individual variability in diel vertical migration of a marine copepod: Why some individuals remain at depth when others migrate. Limnology and Oceanography.

[69] Tarling GA, Jarvis T, Emsley SM, Matthews JBL (2002). Midnight sinking behaviour in Calanus finmarchicus: a response to satiation or krill predation?. Mar. Ecol. Prog. Ser..

[70] Berge J (2014). Arctic complexity: A case study on diel vertical migration of zooplankton. Journal of Plankton Research.

[71] Flögel S, Dullo WC, Pfannkuche O, Kiriakoulakis K, Rüggeberg A (2014). Geochemical and physical constraints for the occurrence of living cold-water corals. Deep-Sea Res. II Top. Stud. Oceanogr..

[72] Price, D. & Davies, A. G. Time-lapse imaging reveals the fine-scale behaviour of Lophelia pertusa polyps in response to changing flow velocity and temperature. *Marine Imaging Workshop, Kiel, Germany* (2017).

[73] Brooke S, Ross SW, Bane JM, Seim HE, Young CM (2013). Temperature tolerance of the deep-sea coral *Lophelia pertusa* from the southeastern United States. Deep-Sea Res. Part II: Top. Stud. in Oceanogr..

[74] Wijgerde, T., Spijkers, P., Karruppannan, E., Verreth, J. A. J. & Osinga, R. Water flow affects zooplankton feeding by the scleractinian coral *calaxea fascicularis* on a polyp and colony level. *Journal of Marine Biology* (2012).

