# Supplementary material for: Computer vision enables short- and long-term analysis of Lophelia pertusa polyp behaviour and colour from an underwater observatory
Source: Sci Rep. 2019 Apr 29;9:6578. doi: 10.1038/s41598-019-41275-1 (PMC6488648; doi:10.1038/s41598-019-41275-1)
Supplement: Supplementary file 1 — Supplementary information [file 41598_2019_41275_MOESM1_ESM.docx]

Supplementary material for

**Computer vision enables short- and long-term analysis of *Lophelia pertusa* polyp behaviour and colour from an underwater observatory**

Jonas Osterloff ^Δ^, Ingunn Nilssen ^*^, Johanna Järnegren ^ω^, Tom Van Engeland^†^, Pål Buhl-Mortensen ^Φ^, Tim W. Nattkemper ^Δ^

^Δ^ Biodata Mining Group, Faculty of Technology, University of Bielefeld, 33501 Bielefeld, Germany

^*^Statoil ASA, research and technology, 7005 Trondheim, Norway

^ω^ Norwegian Institute for Nature Research, P.O. Box 5685 Torgarden, 7485 Trondheim, Norway

^†^ NIOZ Royal Netherlands Institute for Sea Research, Department of Estuarine and Delta Systems, Utrecht University, Netherlands

^Φ^ Research Group Benthic Habitat, Institute of Marine Research, 5817 Bergen, Norway

**S1: Computation of coral colour time series** $\xi_{t}$

The computational method for computing colour features for *Lophelia pertusa* is described. In general, any computational analysis of underwater images requires image pre-processing. Colours in underwater images are affected by object-camera distance and light-source-object distance, as light attenuation is wavelength dependent [44]^[[1]](#footnote-1)^. Furthermore, underwater images often suffer from variation in illumination throughout the image frame due to light attenuation and varying distances to the objects of interest. The physical properties are not only different from imaging in air, they also feature considerable dynamic changes affecting the colours of underwater images. Events such as algae blooms are examples of the latter. The demands for pre-processing of the images are dependent of the requirements for later analysis.

Monitoring of cold water coral (CWC) for colour change using a fixed camera does not require the compensation of colour shifts due to wavelength depended attenuation. Due to the multi spherical structure of a CWC reef, the object-camera and object-illumination distance varies for different objects but is constant over time for non-moving objects. The pre-processing for the colour monitoring (i.e. the mapping of the original image to a colour transformed one ($I_{t}\to{I'}_{t}$)) is therefore reduced to a white balancing of the raw image data with a fixed white balance and a spatial alignment of the photos. To spatially align the images (an algorithmic problem often referred to as image registration) we apply the ECC (Enhanced Correlation Coefficient Maximization) method [46]. In this study a fixed white balance was applied to reduce the colour variance between different images ${(I}_{t}, I_{t+n})$, as white balancing is influenced by the colours of the objects in the visual field. For this fixed white balancing, parameters are computed as the average of the parameters of the auto white balance estimated on a subset of ten images.

Computed parameters were then used for colour transformation of all $I_{t}$. The fixed white balancing was applied to optimize colour constancy over time and the alignment to compensate small camera shifts eventually caused by changing currents. Dynamic changes of the in-optical properties were compensated in the normalization of the extracted live CWC colour (see below).

After pre-processing the images, live coral was segmented in each pre-processed image ${I'}_{t}$ applying machine learning similar to [30]. As proposed in this work, Gabor wavelet responses form the feature space which is clustered using a hierarchical hyperbolic self-organizing map (H^2^SOM) algorithm. In contrast to [30] the selection of H^2^SOM prototypes representing live coral in the feature space, is performed as described in [47], followed by a re-inspection and manual optimization on a small subset of about ten images. In each image ${I'}_{t}$ a binary segmentation (1 = CWC covered pixel, 0 = else) mask $M_{t}^{\mathrm{coral}}$ for the CWC is computed (see upper row in Figure 3). The contours of the segmentation masks differ slightly due to small differences in water qualities or passing fish (i.e. occlusion). From these masks, a CWC mask $\hat{M}^{\mathrm{coral}}$ is created from the intersect of all resulted segmentation masks $M_{t}^{\mathrm{coral}}$. This segmentation approach aims at favoring the segmentation’s precision (i.e. avoiding false positives) over its recall (i.e. avoiding false negatives).

Using this intersect mask $\hat{M}^{\mathrm{coral}}$ the CWC colour is extracted in each image ${I'}_{t}$ as the average coral color in the mask at time point *t*:

|  | ${\bar{\boldsymbol{c}}}_{t}^{\mathrm{coral}}= \frac{1}{w\cdot h}\cdot\sum_{x=1}^{w} \sum_{y=1}^{h} \hat{M}_{\left( x,y \right)}^{\mathrm{coral}}\cdot{I'}_{t,\left( x,y \right)}-{\bar{\boldsymbol{c}}}_{t}^{\mathrm{full}}$, | (1) |
| --- | --- | --- |

with

|  | ${\bar{\boldsymbol{c}}}_{t}^{\mathrm{full}}= \frac{1}{w\cdot h} \cdot\sum_{x=1}^{w} \sum_{y=1}^{h} {I'}_{t,\left( x, y \right)}$. | (2) |
| --- | --- | --- |

as the average colour of the image at time $t$. The last subtraction term in (1) is necessary to compensate colour shifts affecting the visual qualities of all objects in the whole image, e.g. changes of the in-optical properties. Colours ${\bar{\boldsymbol{c}}}_{t}^{\mathrm{coral}}$and ${\bar{\boldsymbol{c}}}_{t}^{\mathrm{full}}$ are extracted using the CIE*Lab* color space [48].

To gain a time series $\xi_{t}$ describing the relevant colour changes of the CWC, we extract the *a*-coordinate from the (*L,a,b*)-color representation (see Figure 3, top right) of ${\bar{\boldsymbol{c}}}_{t}^{\mathrm{coral}}$:

|  | $\xi_{t}={\bar{\boldsymbol{c}}}_{t,a}^{\mathrm{coral}}.$ | (3) |
| --- | --- | --- |

**S2: Computation of coral activity time series** $\gamma_{t}$

The method for computationally estimating the polyp activity in a region of interest is given. First, the images are pre-processed (i.e. all original images are transformed ($I_{t}\to\tilde{I_{t}}$)) quite similar to the method described for colour assessment ($I_{t}\to{I'}_{t}$) above. Again, white balancing with a fixed white balance was applied to the images followed by an image alignment using the ECC method (see above). In contrast to ($I_{t}\to{I'}_{t}$) above, white balancing parameters were chosen manually to increase the contrast for the small morphological polyp details.

To study the temporal dynamics of the CWC polyps’ activities we applied a deep-learning-based computer vision approach in a chosen region of interest (ROI) within the visual field, at each time point *t* to compute the percentage of pixels belonging to active polyps.

In a first step, a ROI of $800 \times600$ pixels in size inside the CWC was selected (see green frame in Figure 3 in the main manuscript). The ROI was selected based on a great proportion of live polyps with good image contrast and sharpness, and a relatively low chromatic aberration and lens-distortion. Next, a gold standard data set was collected representing the visual appearances of coral pixels belonging to active and inactive polyps, respectively. To this end we first conducted a study to examine three experts’ (i.e. the authors JJ, IN, PB) ability of identifying different levels of polyp activity manually in the ROI.

The ROIs from a selected subset of 13 images were inspected using the online annotation system BIIGLE 2.0^^[[2]](#footnote-2)^^ (BioImage Indexing, Graphical Labelling and Exploration 2.0) [49]. The software enables the user to flip quickly between images making the identification of the different levels of polyp activity easier. The annotation of single polyps was performed in two sessions carried out by the three experts individually to collect annotations of sufficient quality. Two annotation sessions were necessary to provide data to estimate inter- and intra-observer agreement of the polyp annotation [50]. In session I, the experts placed circular shaped annotations with different colours for different polyp states. For each annotation, the experts selected a polyp activity level $\omega$. The polyp activity level $\omega$ was chosen from the following classes: “Fully Expanded”, “Fully / Partly Expanded”, “Partly Expanded”, “Partly Expanded / Retracted”, “Retracted”, or “Recently Dead”. So, the first five levels represent the process levels of the polyp reaching from maximum to minimum activity. In session II a subset of three images (of the 13 images) was annotated a second time by the same users (without visualizing the results from session I) after a break of one week.

After both sessions, the different experts' annotations were compared. The data showed that a consistent and reproducible classification of the polyp activity could be achieved, if the six polyp activity levels were fused into just two levels: “active” = {“Fully Expanded”, “Fully / Partly Expanded”, “Partly Expanded”} and “inactive” = {“Partly Expanded / Retracted”, “Retracted”, or “Recently Dead”}. If one polyp was marked by at least two experts and assigned to the same level, i.e. “active” (i.e. class label $\omega=1$) or “inactive” (i.e. class label $\omega=0$), by the experts this position was included as a gold standard annotation $\boldsymbol{g}_{i} = (\left( x, y \right),\omega)$ for *i=1,…,N*. The entire set of all gold standard annotations $\boldsymbol{G=\{}\boldsymbol{g}_{i}\boldsymbol{\}}$ was finally used for training, testing and validation in the machine learning step (see below). Furthermore, the gold standard was used to compute a classification mask $\tilde{M}$ covering the area where polyp activity could successfully be estimated manually. Polyp activity will only be estimated in areas marked positive by$\tilde{M}$.

In order to train, validate and test a deep neural network, image patches of equal size ($46 \times46$) were extracted at the regions defined by each $\boldsymbol{g}_{i}$ summing up to 2410 image patches showing polyps. A background class was generated by extracting 100 image patches at random locations in $\{\tilde{I_{t}}\}$ which were outside the mask $\tilde{M}$. The number of examples was further increased by flipping (augmentation factor: 2x) and rotating (augmentation factor: 12x) the patches and by adding Gaussian noise to them to avoid overfitting (augmentation factor: 2x). Employing these boosting procedures, we achieved a data augmentation by the factor of 48, i.e. an increase of the training data volume by 48-times. Image patches for each machine learning category are divided into training (70 %), validation (20 %) and test (10 %) patches. Training and validation set were used for training and parameter optimization. The test was left out in in this step so it could be used for a final assessment of the network’s performance for new and “unseen” data. A deep convolutional neural network with a LeNet-5 layout [51] is trained on the training patches using the NVIDIA DIGITS software^^[[3]](#footnote-3)^^. The result classifier network constitutes a mapping $C\left( p \right)\to\{0,1\}$ from the input space of image patches to the output space of polyp activity levels. The trained network was evaluated computing the accuracy

$$ACC =\frac{\left| TP \right|+\left| TN \right|}{\left| TP \right|+\left| FP \right|+\left| TN \right|+\left| FN \right|} .$$

The network achieved an accuracy of 0.98 for the training set, 0.96 for the validation set and 0.96 for the test set.

To estimate the polyp activity at one time point *t,* all pixels *(x,y)* inside the mask $\tilde{M}$ are classified to belong to an active polyp or to an inactive*.* To this end, a patch $p_{(x, y)}$ of $46 \times46$ pixels centering in $(x,y)$ is used as input for the trained network and the result classification output $C\left( p_{\left( x, y \right)} \right)$ represents the likelihood of the patch showing an active polyp, i.e. $C\left( p_{\left( x, y \right)} \right)= 1$) or not (i.e. $C\left( p_{\left( x, y \right)} \right)=0$) for retracted polyps or other structures. The aggregated number of pixels classified to be “active” in relation to the marked position in $\tilde{M}$ gives the estimated percentage of active polyps for each sub-image $\tilde{I}_{t}$, referred to as polyp activity $\gamma_{t}$ in the rest of this manuscript:

|  | $\gamma_{t}=\frac{\left\vert\left\{ p_{\left( x, y \right)}\in\tilde{I}_{t} : C\left( p_{\left( x, y \right)} \right)= 1 \right\} \right\vert}{\vert\tilde{M}=1\vert} .$ | (4) |
| --- | --- | --- |

To assess he accuracy of the estimated polyp activity, the results obtained for the 13 images that were evaluated by the human experts were compared to those obtained from the manual classifications. First, the polyp activity was computed for each of the 13 image ROIs using the trained network as a pixel classifier formula (4). Second, for each of the 13 images a reference value *R_t_* for a manually assessed polyp activity was computed, based on the annotations $\boldsymbol{G=}\left\{ \boldsymbol{g}_{i} \right\}$provided by the human experts, so this reference could be used a gold standard in order to correlate the computationally determined polyp activity to the manually determined one. For each image the reference was computed as

$R_{t}=\frac{A^{active}}{|\tilde{M}=1|}$,

with $A^{active}$ as the sum of all areas of annotated active polyps. As all active polyps in the gold standard have been marked by two or three experts (see above) the area $a_{i}$ for one polyp was computed as the union of all circular shaped annotations for this polyp and $A^{active}= \sum_{i} a_{i}$.

**S3: Pearson correlation analysis**

**Table S3**: Pearson correlation coefficients for the estimated polyp activity $\boldsymbol{\gamma}_{\boldsymbol{t}}$ and selected sensor data $\boldsymbol{f}_{\boldsymbol{t}}$ are computed after linear interpolation $\boldsymbol{f}_{\boldsymbol{t}}\boldsymbol{\to}\boldsymbol{x}_{\boldsymbol{t}}$ (first column). Coefficients for the daily averages $\bar{\boldsymbol{\gamma}_{\boldsymbol{t}}}$ and $\bar{\boldsymbol{x}_{\boldsymbol{t}}}$ are given in the second column.

|  | $\boldsymbol{r}\left( \boldsymbol{\gamma}_{\boldsymbol{t}}\boldsymbol{,}\boldsymbol{x}_{\boldsymbol{t}} \right)$ | $\boldsymbol{r}\left( \bar{\boldsymbol{\gamma}_{\boldsymbol{t}}}\boldsymbol{,}\bar{\boldsymbol{x}_{\boldsymbol{t}}} \right)$ |
| --- | --- | --- |
| Conductivity (C) | 0.61 | 0.68 |
| Temperature (T) | 0.48 | 0.54 |
| Salinity (S) | 0.49 | 0.55 |
| Current velocity north (Vn1) | -0.06 | -0.16 |

**S4: Wavelet transformation response visualization (without significance contour plots)**

**
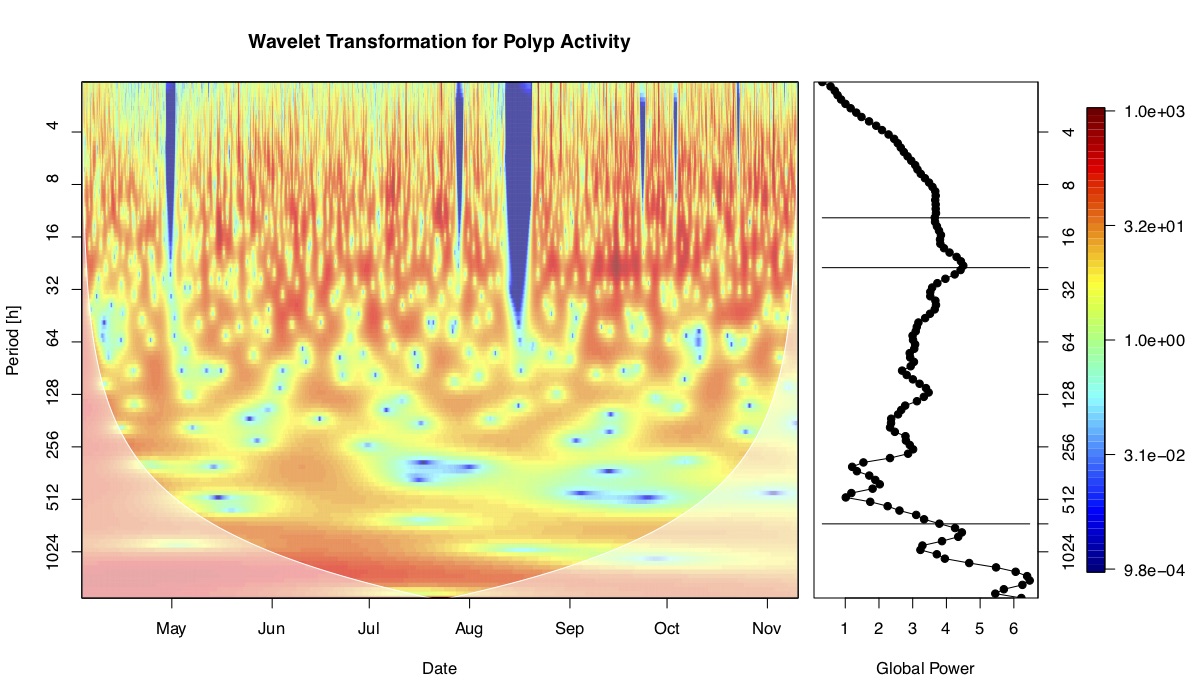
**

Figure S4a: Wavelet transformation response for the polyp activity time series ($\boldsymbol{\gamma}_{\boldsymbol{t}}^{\boldsymbol{'}}$) without black significance contours.

**
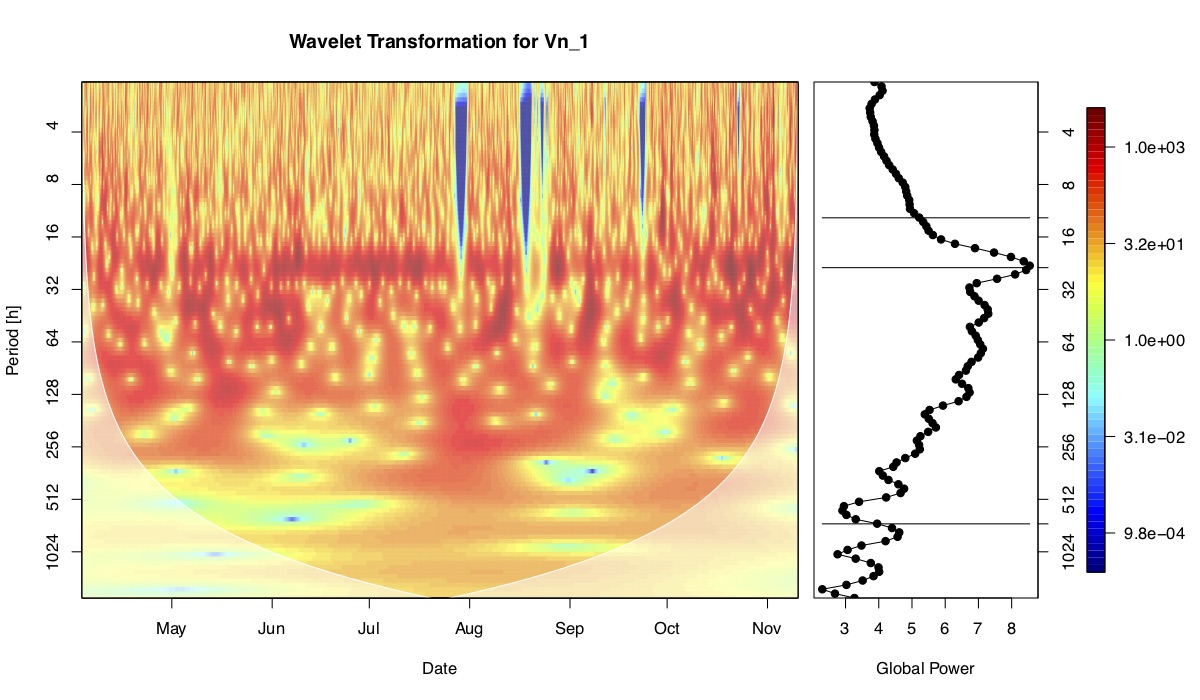
**

Figure S4b: Wavelet transformation response for the current time series (Vn1) without black significance contours.

**S5: Wavelet coherence analysis plot**

**
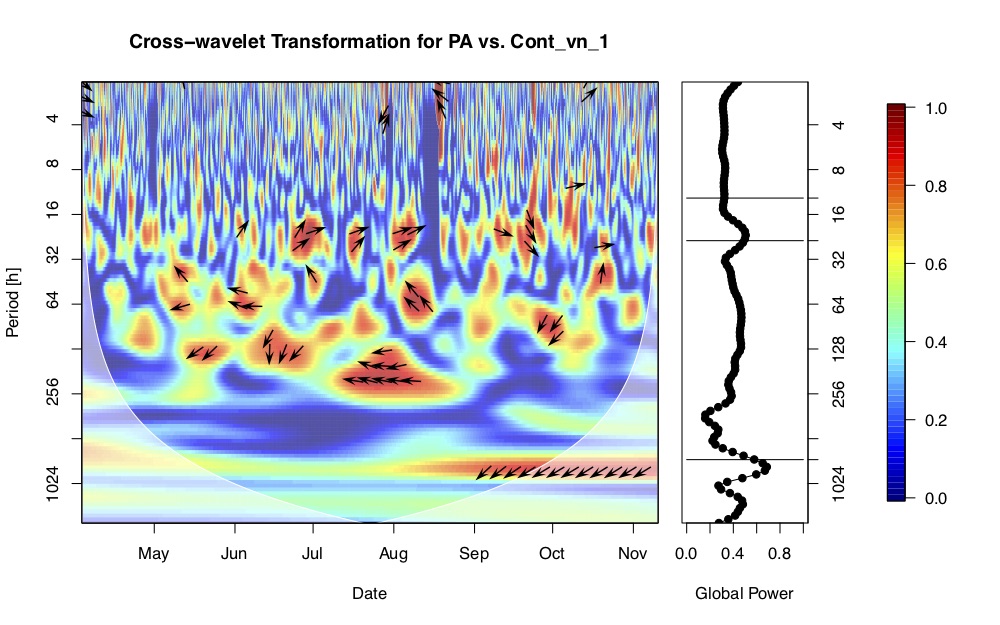
**

Figure S5: Wavelet transformation coherence analysis plot for polyp activity ($\boldsymbol{\gamma}_{\boldsymbol{t}}^{\boldsymbol{'}}$) and current velocity in north direction Vn1

**S6: Polyp activity and current velocity Vn1 for a selected interval of 4 weeks**


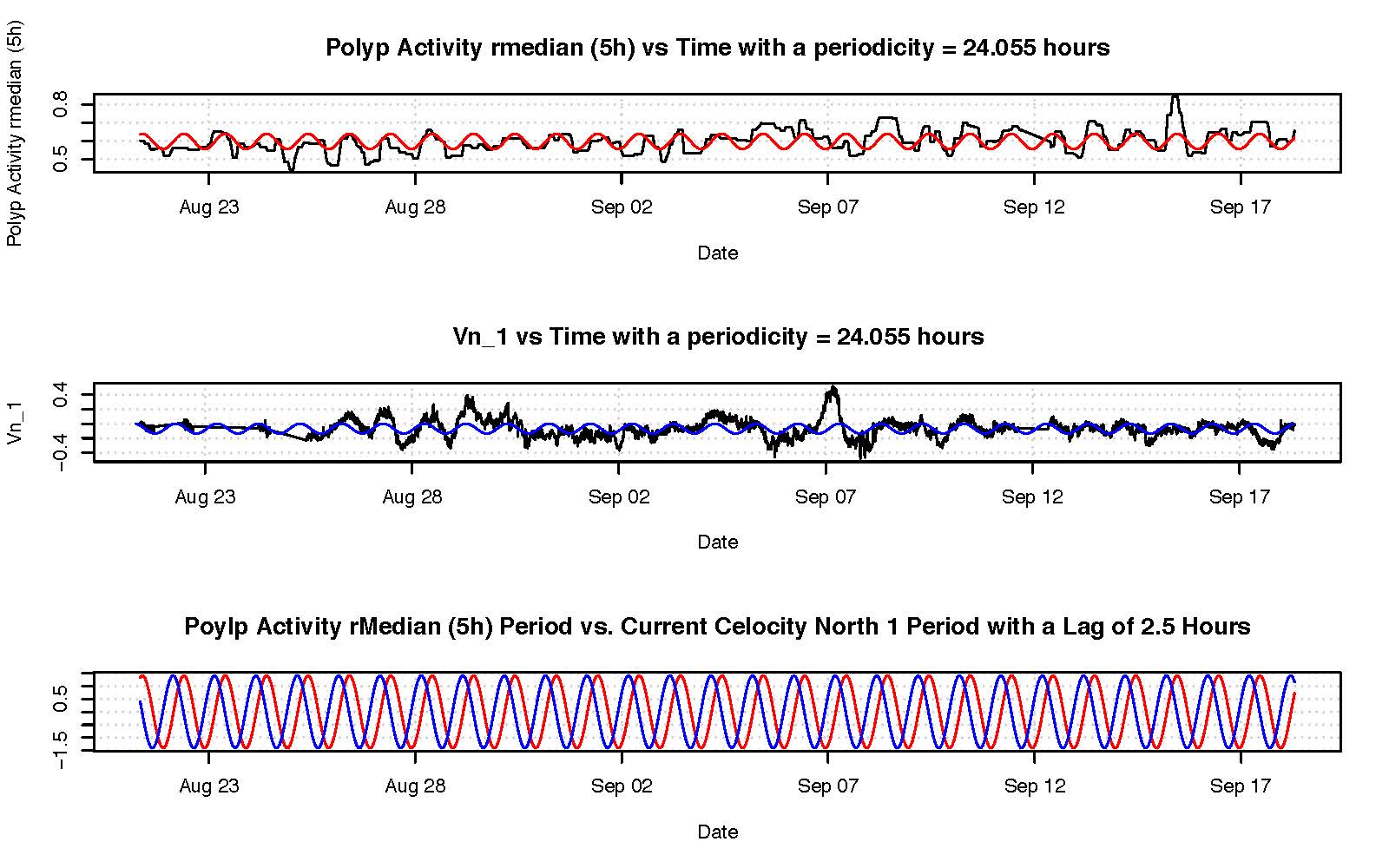


**Figure S6:** Detailed visualization of the coherence between estimated polyp activity $\boldsymbol{\gamma}_{\boldsymbol{t}}$ and measured current velocity in north direction $\boldsymbol{f}_{\boldsymbol{t}}^{\left( \boldsymbol{Vn}\boldsymbol{1} \right)}$ for an interval of four weeks (August 21^st^– September 18^th^, 2015). In the upper row, the running median (5 hours) of $\boldsymbol{\gamma}_{\boldsymbol{t}}$ is plotted (black line) with the corresponding computed periodicity (red line). In the middle row measured $\boldsymbol{f}_{\boldsymbol{t}}^{\left( \boldsymbol{Vn}\boldsymbol{1} \right)}$ is plotted (black line) with the corresponding computed periodicity (blue line). The estimated time lag of 2.5 h between both computed periodicities is illustrated in the lower row.

1. Numbers in the references in the supplementary refer to the references in the main manuscript [↑](#footnote-ref-1)
2. www.biigle.de [↑](#footnote-ref-2)
3. ^https://developer.nvidia.com/digits^ [↑](#footnote-ref-3)
